# Comparison of HTA Appraisal Outcomes in Japan, the UK, and Canada: A Cross-Sectional Mapping Study

**DOI:** 10.36469/001c.172102

**Published:** 2026-11-13

**Authors:** Koichi Tsuda, Asuka Suzuki, Yoshinori Nakata

**Affiliations:** 1 Teikyo University Graduate School of Public Health, Tokyo, Japan; 2 argenx Japan K.K., Tokyo, Japan

**Keywords:** reimbursement, drug pricing, NICE, CDA-AMC, Japan, cost-effectiveness evaluation, health technology assessment

## Abstract

**Background:**

Japan’s cost-effectiveness evaluation (CEE) system is a health technology assessment (HTA) framework used to inform post-listing price adjustments. However, comparisons of its appraisal outputs with those of coverage-oriented HTA systems in the UK and Canada remain limited.

**Objectives:**

To describe the distribution and agreement of study-defined categories assigned to HTA appraisal outputs in Japan, the UK, and Canada for Japan’s CEE-evaluated products, and to describe CEE-derived price changes in Japan by additional clinical benefit status.

**Methods:**

We mapped published appraisal outputs for 43 CEE-evaluated products to 4 common categories (Positive, Conditional, Negative, Not assessed) using CEE price-adjustment decisions in Japan, appraisal or access decisions in the UK, and reimbursement recommendations in Canada. For the main analysis, only final decisions were eligible; products without one were classified as Not assessed. Direct UK-Canada population alignment was assessed post hoc.

**Results:**

Conditional predominated (Japan 79.1%, UK 55.8%, Canada 65.1%), and Not assessed accounted for 23.3% in the UK and 25.6% in Canada; Negative was not applicable in Japan’s CEE framework, and no Canadian product was classified as Positive. Complete 3-jurisdiction agreement occurred in 14/43 products (32.6%) and pairwise agreement ranged from 41.9% to 60.5%; after excluding Not assessed classifications, pairwise agreement was 54.5% to 68.8%, and 3-jurisdiction agreement was 48.3%. Among pairwise comparisons, only UK-Canada exceeded the agreement expected under independent classification (60.5% vs 42.7%). Of the 19 concordant products in the direct UK-Canada alignment analysis, only 1 had fully aligned appraisal populations. The median CEE-derived price change was −4.28%; by additional clinical benefit status, medians were −1.43% (demonstrated, n = 21), −4.28% (mixed, n = 16), and −6.01% (not demonstrated, n = 6).

**Discussion:**

The institutional meaning of Conditional differed: price adjustment in Japan, clinical restrictions or managed access in the UK, and reimbursement recommendations with clinical and/or pricing conditions in Canada. Mapped-category agreement should not be interpreted as evidence of similarity between HTA systems.

**Conclusions:**

Mapped-category agreement coexisted with differences in institutional meaning and appraised populations. In Japan, price changes varied by additional clinical benefit status, but these differences should be interpreted under price-adjustment rules that themselves incorporate assessments of additional clinical benefit.

## BACKGROUND

Japan’s cost-effectiveness evaluation (CEE) system became fully operational in April 2019, establishing a price-adjustment-oriented health technology assessment (HTA) framework^1–3^ in which incremental cost-effectiveness ratio classifications and evaluation results are published and may be reflected in drug price adjustments under the National Health Insurance (NHI) system.^2,4–6^ Unlike many high-income countries where confidential discounts may create differences between publicly listed and actual negotiated prices, Japan maintains nationally published NHI reimbursement prices.^2,4–6^ Because international reference-pricing approaches use observable prices in other countries as benchmarks, changes in Japan’s publicly listed NHI prices may also be relevant beyond the domestic reimbursement system.^7^

Japan’s CEE system has recently undergone reform and remains under review. Drawing on operational experience since 2019, the Ministry of Health, Labour and Welfare (MHLW) has indicated a policy direction to expand the scope of price adjustment for drugs that lack additional clinical benefit while adding to costs, as reflected in the ministerial budget agreement of December 2025.^8^

The institutional role and consequence of appraisal differ across the jurisdictions compared here.^9^ In Japan, CEE is conducted after NHI listing and informs price adjustment while reimbursement is maintained.^3,4^ In the UK comparator used in this study, decisions by the National Institute for Health and Care Excellence (NICE) and National Health Service (NHS) England inform whether and on what terms a technology is used in the NHS. Canada’s Drug Agency (CDA-AMC; formerly CADTH) issues reimbursement recommendations to participating public drug plans; these recommendations inform subsequent price negotiation and listing decisions. Comparison using common operational categories may therefore characterize published appraisal outputs without assuming equivalence between the underlying decision processes.

Prior research has addressed cross-national HTA comparisons and Japan’s CEE system from several perspectives. HTA decisions have been compared across the UK, Canada, Australia, and other jurisdictions, including for orphan drugs approved in Japan.^10–14^ Within Japan’s CEE system, discrepancies between company-submitted and public analyses have been reported, although CEE-based price adjustment rates were explicitly excluded from that analysis.^15^ No prior study has both systematically mapped appraisal outputs from Japan, the UK, and Canada to common categories and quantitatively compared their distribution and mapped-category agreement using Japan’s CEE-evaluated products as the index cohort.

The primary objective was to map published HTA appraisal outputs in Japan, the UK, and Canada for Japan’s CEE-evaluated products to study-defined categories and to describe the resulting category distributions and cross-jurisdictional agreement. The secondary objective was to describe CEE-derived NHI drug price changes by product-level additional clinical benefit status derived from the published public analysis findings.

## METHODS

### Study Design and Eligibility

We conducted a cross-sectional descriptive study of products designated for CEE under Japan’s CEE framework. Japan’s CEE designation categories are labeled H1-H5. H1 and H2 denote newly listed products with predicted peak annual sales of at least ¥10 billion and ¥5 billion to less than ¥10 billion, respectively; H3 covers specified products with an exceptionally high reimbursement price, indications added after determination of the analytic framework, or new evidence judged to materially affect the evaluation; H4 applies to already listed products with annual sales of at least ¥100 billion or meeting the exceptional-price or postevaluation new-evidence criteria; and H5 groups products with a reference product and applies its evaluation result without independent cost-effectiveness analysis.^2^ The UK and Canada were selected as comparator jurisdictions because both have established coverage-oriented HTA systems^9^ and publish appraisal or reimbursement decisions and supporting economic evaluations that permit reproducible product-level comparison. Products were excluded if they were (1) medical devices, (2) designated under the H5 category, (3) subject to a discontinued analysis, or (4) without a confirmed evaluation result at the data cutoff (May 31, 2026). The unit of analysis was the drug product, with one mapped category per product-jurisdiction pair. Products with a single product-level CEE evaluation result were retained as product-level units even when the CEE target encompassed multiple indications.

Products and cross-jurisdictional appraisal decisions were identified as of May 31, 2026. For the fixed eligible cohort, publicly announced post-CEE adjusted NHI prices were tracked through August 5, 2026. An adjusted price was considered available once the price-adjustment decision and revised NHI price had been publicly announced, regardless of the effective date.

Final decisions explicitly superseded or replaced by the issuing agency were not eligible for primary selection but were retained as historical supporting sources. Where multiple eligible final decisions existed, one exactly matching the Japan CEE target indication and population was selected when available. When the Japan CEE target encompassed multiple indications, an appraisal addressing only one indication could qualify as an Exact match at the indication level but not at the product level. When no single appraisal covered the full product-level target, one eligible appraisal was designated as the primary source under the selection hierarchy, with additional indication-specific appraisals retained as supporting sources in the audit trail.

If no eligible Exact match existed, the decision with the greatest clinically meaningful overlap across 7 dimensions (disease or histologic subtype, biomarker, severity or disease activity, age, prior treatment, line of therapy, and treatment regimen) was selected, with particular attention to disease or histologic subtype and line of therapy where clinically applicable. No fixed numerical weights or composite scores were applied; differences were assessed for clinical materiality in the context of each condition. If multiple decisions remained equally suitable, the most recent was selected. Recommendation favorability was not considered in source selection. Alternative candidates and selection rationales are documented in the **Supplementary Data File**, with material cases described in **Supplementary Notes S.N.1-S.N.5.**

### Data Sources and Data Extraction

**Japan**: Central Social Insurance Medical Council (Chuikyo) plenary documents on CEE-based price adjustments^16^ and publicly available materials of the Center for Outcomes Research and Economic Evaluation for Health (C2H), including company-submitted analyses and public reanalyses.^17^

**United Kingdom:** NICE Technology Appraisals (TAs) and related documentation. Under the study’s operational source hierarchy, when no eligible final TA corresponding to the Japan CEE target was available, a formally published NICE Guideline (NG) or NHS England commissioning policy—although not a formal TA—was included as an appraisal-equivalent access decision and mapped using the same classification criteria.^18^ Here, “UK” refers operationally to decisions issued by NICE and NHS England, and not to the separate processes of the Scottish Medicines Consortium or the health service in Northern Ireland.

**Canada**: Reimbursement recommendations and related economic review documents issued by CDA-AMC.^19,20^ The NICE and CDA-AMC websites were searched using generic and brand names. Agency product pages and linked appraisal or reimbursement-review documents were reviewed to identify all potentially eligible indication-specific decisions, confirm whether final guidance or recommendations had been published by the data cutoff, and document the status of ongoing, terminated, cancelled, or otherwise incomplete reviews.

Product-level classifications and source-level documentation, including document identifiers, decision dates, indication/population alignment, and mapping rationale, are provided in the Supplementary Data File (Excel file). Alignment of each UK or Canadian appraisal with the Japan CEE target (Japan-anchored alignment) was assessed across these 7 dimensions. Overall alignment was classified as Exact when all clinically material dimensions matched, Partial when clinically meaningful overlap remained despite at least one material difference, Closest available when no sufficiently matching appraisal existed and the eligible appraisal with the greatest clinically meaningful overlap was used, or No match/Not evaluated when no corresponding eligible appraisal was identified.

Product characteristics comprised CEE designation category, orphan and/or intractable disease designation, and therapeutic area. These characteristics were obtained from C2H and Chuikyo records, with therapeutic area assigned based on the principal Japan CEE target indication. Appraisal sources and decision characteristics were extracted using a structured data-extraction form (**Supplementary Appendix**). The mapping rules, product-level classifications, and selected boundary cases were reviewed with the coauthors. Boundary cases were products for which a documented alternative source-selection or decision-status interpretation—such as exact-indication matching, an alternative eligible source or population-specific decision, or inclusion of a draft document—could have changed the mapped category; they were resolved by applying the documented rules and are recorded individually in the source-level audit trail.

### Common Appraisal Categories

Japan’s CEE informs price adjustment after NHI listing rather than insurance coverage,^3,4^ whereas UK decisions inform NHS use and Canadian recommendations inform reimbursement decisions; both may also reflect considerations beyond cost-effectiveness analysis. The 4 categories defined below therefore represent an operational harmonization for descriptive cross-jurisdictional comparison, not institutionally equivalent decision states; agreement between them should be read as concordance in study-defined mapped categories rather than similarity in institutional function, decision thresholds, underlying evidence, or populations appraised.

Each appraisal output was mapped to 1 of 4 mutually exclusive categories: (A) Positive—recommendation or reimbursement without appraisal-derived additional clinical use restrictions for the evaluated indication or principal target population, or, in Japan, no CEE-based price reduction; (B) Conditional—recommendation, reimbursement, or continued access with appraisal-derived clinical conditions, subgroup restrictions, managed or temporary access arrangements, or a pricing condition or price-adjustment decision forming part of the formal appraisal output in Japan or Canada; (C) Negative—non-recommendation or reimbursement refusal for routine clinical use, including recommendations limiting use to research settings only; and (D) Not assessed—no evaluation conducted, evaluation ongoing, evaluation incomplete due to manufacturer withdrawal or failure to submit evidence, or procedural incompletion (eg, pending appeal outcome or additional committee review) resulting in no published final guidance or final recommendation at the data cutoff.

Jurisdiction-specific mapping rules were as follows. Japan (CEE): no CEE-based price reduction → Positive; CEE-based price reduction with continued NHI reimbursement → Conditional—indicating only that the formal appraisal altered the product’s post-listing price while reimbursement continued, not that the decision was equivalent to a conditional reimbursement or access decision in the UK or Canada; Negative is not applicable because the framework produces no negative appraisal output and NHI reimbursement is maintained after CEE, so a CEE-based price reduction was not mapped to Negative. United Kingdom (NICE and NHS England; main analysis): an eligible final decision recommending use without appraisal-derived restrictions → Positive; a recommendation with appraisal-derived restrictions (subgroup, severity, line of therapy, stopping rule), an optimized recommendation, or managed access (Cancer Drugs Fund, Innovative Medicines Fund, or managed access arrangement) → Conditional; a final non-recommendation → Negative; and no eligible final appraisal-equivalent decision at the data cutoff → Not assessed. The same boundary criteria were applied to the NICE Guidelines and NHS England commissioning policies included in the main analysis.

For Canada (CDA-AMC): Reimburse (unconditional) → Positive; Reimburse with clinical criteria and/or conditions → Conditional; Do not reimburse/Do not list → Negative; and no published final recommendation at the data cutoff → Not assessed.

Restrictions inherent in the marketing authorization were not counted as appraisal-derived restrictions. Price-related arrangements were classified according to their role in each jurisdiction’s formal decision output rather than their mere presence. In the UK, commercial arrangements alone were not treated as appraisal-derived clinical use restrictions and did not change an otherwise Positive classification. In contrast, a pricing condition explicitly incorporated into a CDA-AMC reimbursement recommendation contributed to classification as Conditional.

The main analysis included only final NICE guidance, NHS England commissioning policies, and CDA-AMC recommendations; draft documents were considered only in supplementary boundary-case assessments. Cross-jurisdictional mapped-category agreement was evaluated as the proportion of products with complete 3-jurisdiction agreement, the proportion with disagreement in at least one jurisdiction, and pairwise agreement rates. Because the objective was descriptive, agreement was summarized as unweighted simple categorical agreement. Chance-corrected coefficients such as kappa were not reported because category availability differed structurally across jurisdictions (eg, no Negative category in Japan), a condition under which such coefficients can behave paradoxically. Because imbalanced marginal distributions can instead make simple agreement appear high, each observed rate was reported alongside the agreement expected under independent classification and the maximum agreement attainable given the observed marginal distributions.

### Sensitivity and Secondary Analyses

For the UK, a TA-only strict sensitivity analysis restricted the appraisal pool to NICE Technology Appraisals, excluding NICE Guidelines and NHS England commissioning policies, to assess the impact of the main-analysis definition on category distribution. Where an exact-match NICE appraisal had been terminated and was therefore ineligible for the main analysis, the eligible final NICE decision with the greatest clinically meaningful overlap for the same drug and disease area was selected, with recency used only as a tie-breaker. The classification under exact-indication matching was examined as a boundary-case assessment. Classification changes under a draft-document-inclusive scenario were similarly described for products with final guidance or recommendations pending at the data cutoff, and alternative eligible source selections were examined for material multi-decision boundary cases.

Pairwise agreement was also recalculated after excluding products classified as Not assessed in either jurisdiction of each comparison. Complete 3-jurisdiction agreement was likewise recalculated among products without a Not assessed classification in any jurisdiction. These sensitivity and boundary-case analyses are summarized in **Supplementary Tables S1-S3**, with product-specific boundary cases detailed in **Supplementary Notes S.N.1-S.N.5**. Among products with concordant UK-Canada classifications, the distribution of mapped categories in Japan was also examined.

In exploratory sensitivity analyses, Japanese products with CEE-derived price reductions smaller than 2% or 5% in magnitude, respectively, were reclassified from Conditional to Positive, while classifications in the UK and Canada were unchanged. These were analytical materiality thresholds and do not represent formal thresholds in Japan’s CEE framework. Agreement estimates were recalculated under each threshold (**Supplementary Table S4**).

As an exploratory post hoc analysis, we assessed how closely the populations addressed by the selected UK and Canadian decisions corresponded to each other, rather than to the Japan CEE target. The decisions compared were those selected for the Japan-anchored main analysis; they were not reselected to maximize bilateral population overlap.

Products with an eligible final decision in both jurisdictions under the main-analysis source definitions were classified as comparable; the remainder were classified as not comparable and were not assigned a direct alignment category. For comparable products, the same 7 dimensions used in the Japan-anchored alignment assessment were applied directly to the selected UK and Canadian decision populations. Dimension-level findings were recorded as Same, Different, Not applicable, or Unclear, and overall alignment as Exact, Partial, Minimal or no clinically meaningful overlap, or Unclear. This analysis was descriptive and was not performed in duplicate. Product-level classifications are provided in the **Supplementary Data File**. Here, Exact and Partial refer to alignment between the selected UK and Canadian decision populations, rather than alignment with the Japan CEE target used in the Japan-anchored assessment.

As a secondary analysis, we calculated the CEE-derived NHI drug price change for all 43 products. Pre- and post-adjustment prices were the NHI prices immediately before and after the CEE-based adjustment, as reported in Chuikyo and MHLW materials, using the same strength and package presentation. Price change was defined as (post-adjustment price − pre-adjustment price)/pre-adjustment price × 100. As initially implemented, CEE-based adjustment applied to a defined portion of the price, and the adjustment rate was set stepwise by the incremental cost-effectiveness ratio (ICER): reference values were increased 1.5-fold for oncology, pediatric, and designated intractable or rare disease products, price reduction stopped at the price corresponding to an ICER of ¥5 million (¥7.5 million for those products) per quality-adjusted life-year (QALY), and the reduction was capped at 10% to 15% of the pre-adjustment price; an upward-adjustment scheme also exists.^3,4^ By the end of the study period, for populations without demonstrated additional clinical benefit in the cost-increase category, the prevailing rule applied a coefficient to the usefulness-premium component of the price; this approach was under revision.^2^

Additional clinical benefit findings were extracted at the analysis-population level from C2H public analysis documents using the official reporting categories.^21,22^ These findings were then aggregated into a product-level status: demonstrated in all evaluated populations; not demonstrated in any; mixed, defined as demonstrated in some but not all evaluated populations, including cases in which findings for the remaining populations were not assessable; or not assessable (C2H result unavailable or inconclusive). Descriptive summaries of CEE-derived price changes were calculated for the 3 observed product-level categories: demonstrated, mixed, and not demonstrated. In a post hoc descriptive stratification, median price changes were also summarized among products that underwent a CEE-derived price reduction. All analyses were descriptive. Calculations and tabulations were performed in Microsoft Excel, and figures were generated using Python 3.12 with matplotlib 3.10.

## RESULTS

### Study Cohort and Product Characteristics

Of the 75 products initially designated for CEE, 32 were excluded: 3 medical devices, 13 H5-category products, 1 with a discontinued analysis, and 15 without a confirmed evaluation result at the data cutoff. The remaining 43 products comprised the main analysis cohort (**Figure 1**). Most products, 37/43 (86.0%), were designated H1; 12 products (27.9%) had orphan or intractable disease designations, and oncology was the most frequent therapeutic area (12 products, 27.9%) (**Supplementary Table S5**).

**Figure 1.**
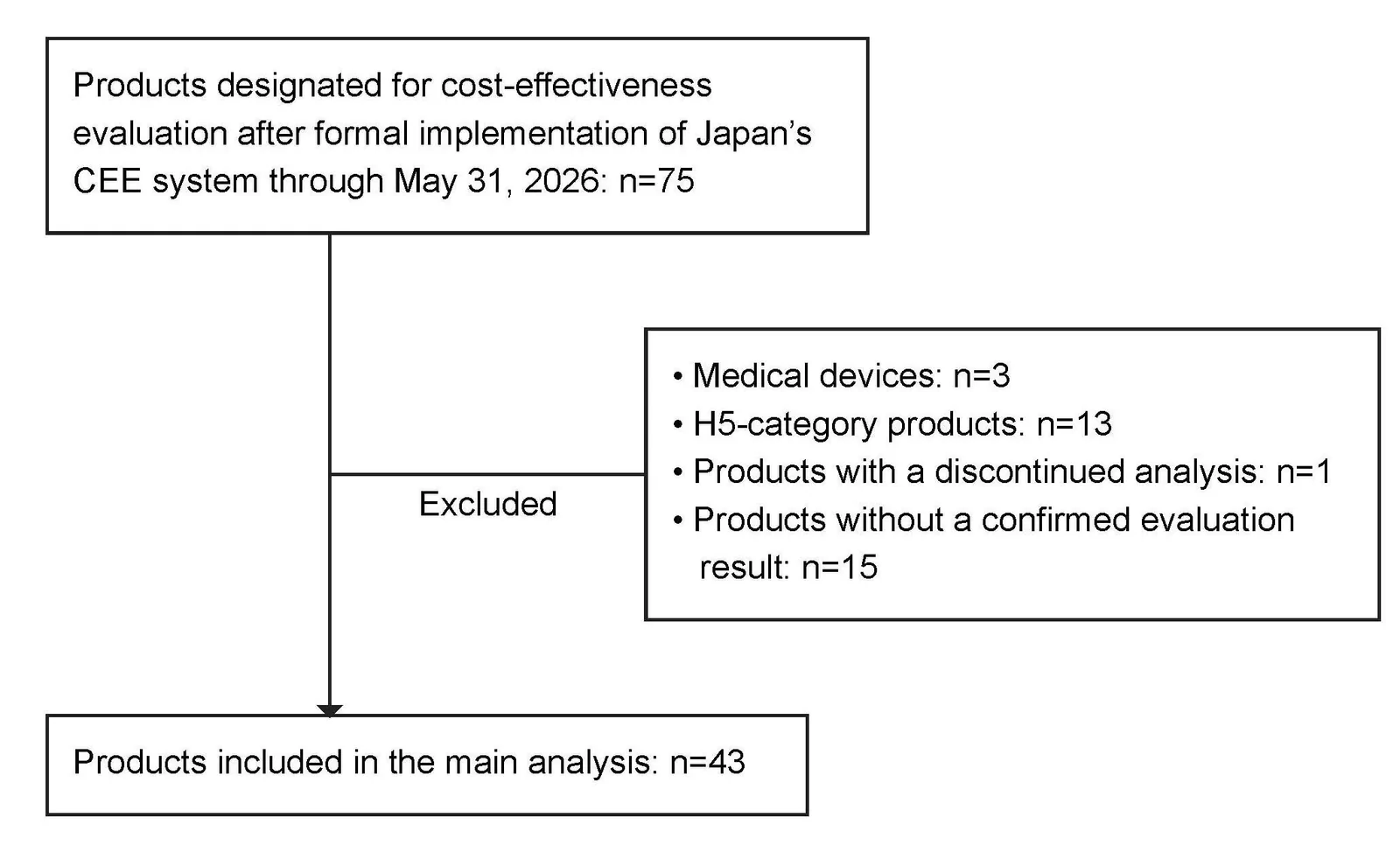
Product Selection Flowchart for the Study Of the 75 products designated for cost-effectiveness evaluation, 32 were excluded, leaving 43 products in the main analysis cohort.

### Common Appraisal Category Distributions and Cross-Jurisdictional Mapped-Category Agreement

In the main analysis, the mapped Conditional category was the most frequent in all 3 jurisdictions: Japan 34/43 (79.1%), UK 24/43 (55.8%), and Canada 28/43 (65.1%); Not assessed accounted for 10/43 (23.3%) in the UK and 11/43 (25.6%) in Canada (**Figure 2**). Classifications, source details, and alignment assessments are provided in the **Supplementary Data File**.

**Figure 2.**
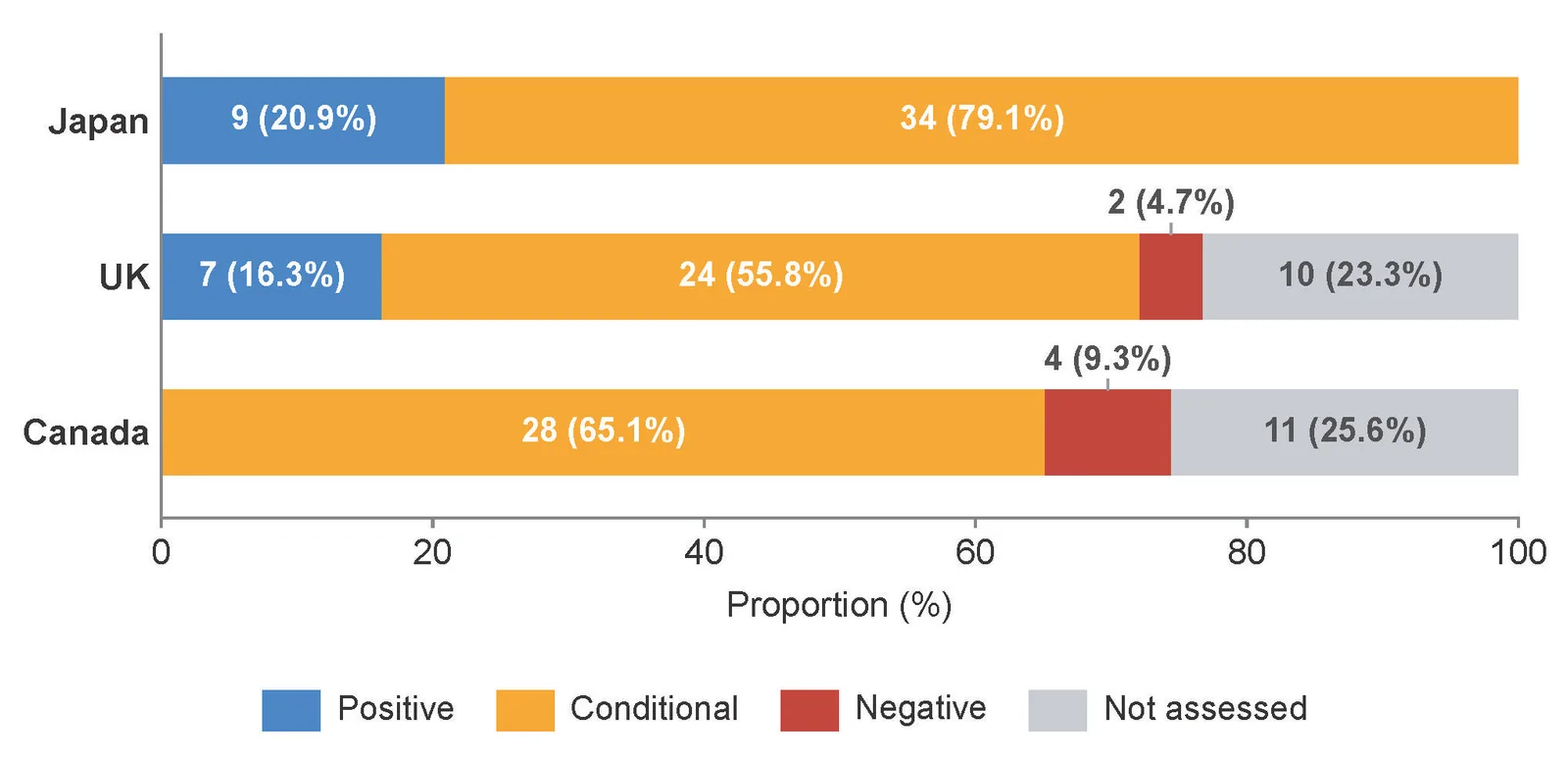
Distribution of Mapped Appraisal Categories Across Japan, the UK, and Canada Under the Main Analysis Bars show the proportions of the 43 included products mapped to each of the 4 common appraisal categories in each jurisdiction.

The current Japanese framework does not provide for a Negative appraisal output; accordingly, all evaluated products were classified as Positive (no CEE-based price reduction) or Conditional (CEE-derived price reduction). In Canada, no product met the operational definition of unconditional Positive: all favorable final CDA-AMC recommendations in this sample included clinical and/or price conditions. UK Conditional classifications reflected subgroup or line-of-therapy restrictions, stopping rules, or managed access through the Cancer Drugs Fund, Innovative Medicines Fund, or a managed access agreement. The operational content of the Conditional classification differed across jurisdictions (**Table 1**).

**Table 1.** Jurisdiction-Specific Meaning and Implications of the Conditional Category

**Jurisdiction**	**Conditional, n (%)**	**Operational Basis for Conditional**	**Institutional Implication**
Japan	34/43 (79.1)	CEE-based price reduction (observed reductions, −15.00% to −0.16%)	NHI reimbursement maintained; listed NHI price reduced through CEE-based adjustment
UK	24/43 (55.8)	Appraisal-derived clinical restrictions or managed access	NHS use restricted by population or treatment conditions, or provided through managed access arrangements
Canada	28/43 (65.1)	Recommendation to reimburse with clinical and/or pricing conditions	Recommendation informs subsequent price negotiation and public drug plan listing decisions

Abbreviations: CDA-AMC, Canada’s Drug Agency; CEE, cost-effectiveness evaluation; NHI, National Health Insurance; NHS, National Health Service.

In the UK, commercial pricing arrangements alone did not result in classification as Conditional. CDA-AMC recommendations do not themselves determine final public drug plan listing or realized patient access. Percentages are of the 43 included products. Nine Japanese products had no CEE-based price change and were classified as Positive.

The 10 UK Not assessed products reflected absent, terminated, or nonfinal appraisals. The 11 Canadian Not assessed products included 6 with no eligible review, 1 with a cancelled regulatory submission, and 4 with ongoing or nonfinal reviews; lecanemab was Not assessed in both jurisdictions (**Supplementary Note S.N.2**). The products classified as Negative were efgartigimod alfa and zolbetuximab in the UK, and posaconazole, andexanet alfa, deucravacitinib, and teprotumumab in Canada. All remained reimbursed under Japan’s NHI system and were classified as Positive or Conditional.

Indication/population scope alignment, assessed separately from final-decision eligibility, was Exact for only 2/43 products (4.7%) in each comparator jurisdiction. Partial was the predominant classification (UK, 36/43 [83.7%]; Canada, 34/43 [79.1%]); 1 UK product was classified as Closest available, while 4 UK and 7 Canadian products were classified as No match/Not evaluated. In each comparator jurisdiction, one of the two Exact matches lacked an eligible final decision: the matching NICE appraisal for gefapixant was terminated, whereas no final Canadian recommendation for ritlecitinib had been published at the data cutoff. Consequently, only 1/43 products (2.3%) in each comparator jurisdiction had both Exact alignment and an eligible final decision.

In the main analysis, complete 3-jurisdiction mapped-category agreement was observed for 14/43 products (32.6%), and all 3 jurisdictions had different mapped categories for 5/43 (11.6%). Among the 24 products for which exactly 2 jurisdictions were concordant, the UK and Canada were concordant for 12, Japan and Canada for 8, and Japan and the UK for 4 (**Figure 3**). Pairwise agreement was 26/43 (60.5%) for the UK-Canada comparison, 22/43 (51.2%) for Japan-Canada, and 18/43 (41.9%) for Japan-UK. Because Conditional was the only mapped category represented in all 3 jurisdictions in this cohort, complete 3-jurisdiction agreement could arise only in that category; all 14 products with complete agreement were Conditional. Observed agreement exceeded the agreement expected under independent classification for the UK-Canada and 3-jurisdiction comparisons, was close to it for Japan-Canada, and was below it for Japan-UK; the maximum attainable agreement also differed across comparisons (**Table 2**). Among the 26 products with concordant UK-Canada classifications, Japan had the same mapped category in 14 (53.8%). Of the 12 for which Japan differed, 7 were categorized as Not assessed in both comparator jurisdictions—a category unavailable to Japan under the study design—and the remaining 5 were Conditional in both but Positive in Japan. Among the 19 products with concordant UK-Canada Conditional classifications, Japan was also Conditional in 14 (73.7%).

**Table 2.** Observed, Expected, and Maximum Attainable Cross-Jurisdictional Mapped-Category Agreement in the Main Analysis

**Comparison**	**Observed Agreement, n (%)**	**Expected Under Independence, %**	**Attainable Maximum, n (%)**
Japan-UK	18/43 (41.9)	47.5	31/43 (72.1)
Japan-Canada	22/43 (51.2)	51.5	28/43 (65.1)
UK-Canada	26/43 (60.5)	42.7	36/43 (83.7)
All 3 jurisdictions	14/43 (32.6)	28.7	24/43 (55.8)

Expected agreement was calculated under independent classification from the observed marginal distributions in each jurisdiction, as the sum across categories of the products of the jurisdiction-specific marginal proportions (the sum of triple products for the 3-jurisdiction comparison); the attainable maximum is the sum across categories of the smallest jurisdiction-specific frequency. It is a ceiling determined by the observed marginal distributions rather than a target. Japan’s framework produces no Negative and, by cohort definition, no Not assessed classifications, and no Canadian product met the operational definition of unconditional Positive in this cohort; Conditional was therefore the only category represented in all 3 jurisdictions.

**Figure 3.**
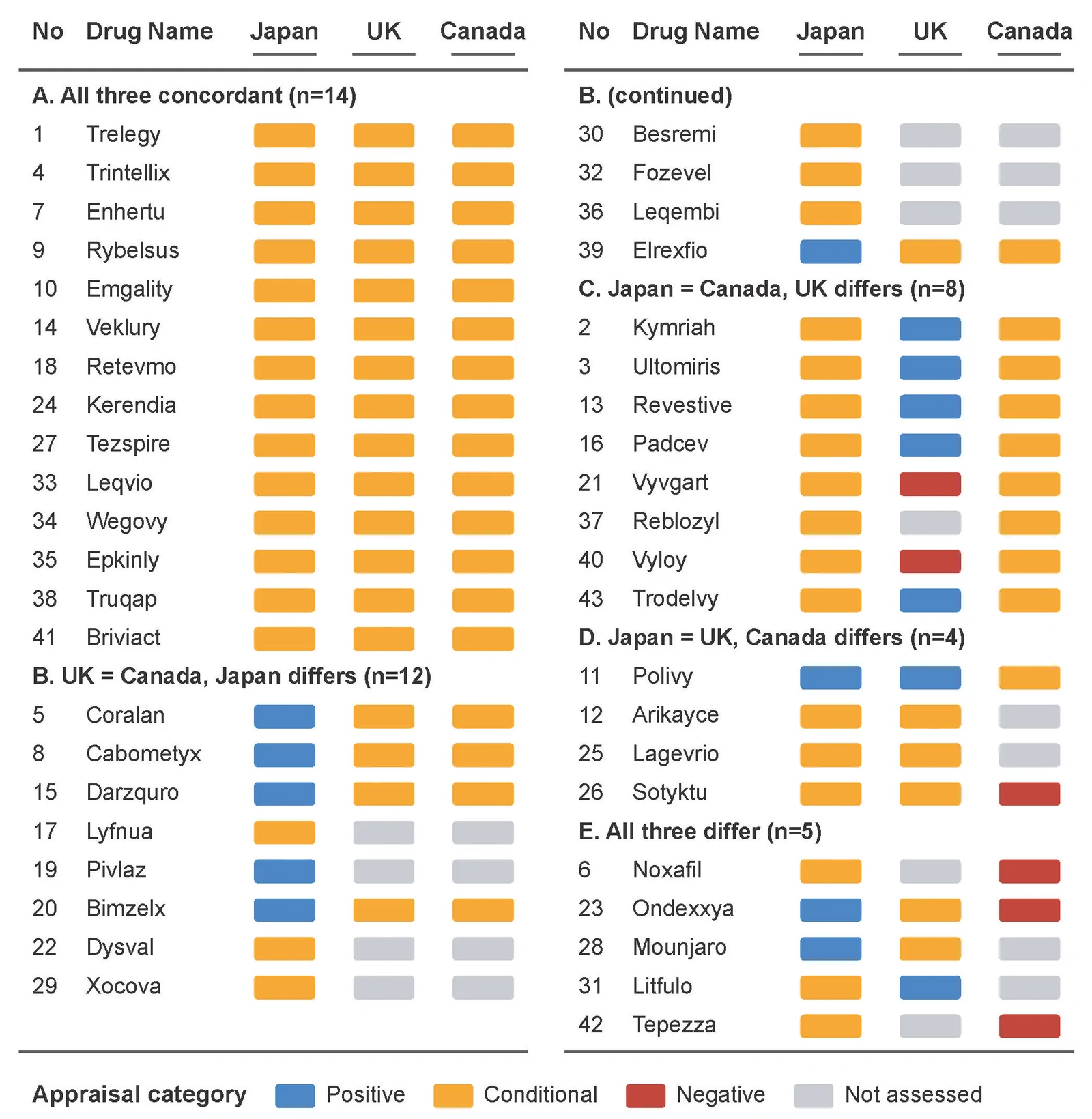
Product-Level Map of Common Appraisal Categories Across Japan, the UK, and Canada Each colored cell represents the mapped appraisal category for one product-jurisdiction pair among the 43 included products. Products are ordered by mapped-category agreement pattern rather than by product number: **A**, concordant in all 3 jurisdictions (n = 14); **B**, UK and Canada concordant and Japan different (n = 12); **C**, Japan and Canada concordant and the UK different (n = 8); **D**, Japan and the UK concordant and Canada different (n = 4); and **E**, all 3 jurisdictions different (n = 5). Within each block, products are ordered by product number. Product numbers correspond to those in the **Supplementary Data File.**

### Cross-Jurisdictional Sensitivity and Boundary-Case Assessments

In the TA-only strict sensitivity analysis, 4 UK products were reclassified from Conditional to Not assessed, reducing UK Conditional classifications from 24/43 (55.8%) to 20/43 (46.5%), although Conditional remained the most frequent UK category. Japan-UK agreement decreased from 18/43 (41.9%) to 14/43 (32.6%), UK-Canada agreement from 26/43 (60.5%) to 24/43 (55.8%), and complete 3-jurisdiction agreement from 14/43 (32.6%) to 11/43 (25.6%); Japan-Canada agreement was unchanged at 22/43 (51.2%) (**Supplementary Tables S1 and S2**). These 4 products had no eligible final NICE Technology Appraisal corresponding to the Japan CEE target and were classified in the main analysis using a NICE Guideline or NHS England commissioning policy. Under the TA-only restriction, they were therefore reclassified as Not assessed; this reflected the source-eligibility definition rather than a change in the underlying UK access position.

In the complete-case sensitivity analysis excluding Not assessed classifications, agreement was 18/33 (54.5%) for Japan-UK, 22/32 (68.8%) for Japan-Canada, and 19/29 (65.5%) for UK-Canada; complete 3-jurisdiction agreement was 14/29 (48.3%) (**Supplementary Table S3**).

Across product-specific boundary-case scenarios, no agreement estimate changed by more than 2.4 percentage points; detailed results and classification rationales are provided in **Supplementary Table S2** and **Supplementary Notes S.N.1-S.N.5**.

Under the exploratory materiality thresholds, Japanese Conditional classifications decreased from 34/43 (79.1%) to 30/43 (69.8%) at the 2% threshold and to 17/43 (39.5%) at the 5% threshold. Japan-UK agreement decreased from 18/43 (41.9%) to 16/43 (37.2%) and 11/43 (25.6%), Japan-Canada agreement from 22/43 (51.2%) to 20/43 (46.5%) and 9/43 (20.9%), and complete 3-jurisdiction agreement from 14/43 (32.6%) to 12/43 (27.9%) and 4/43 (9.3%), respectively; UK-Canada agreement was unaffected because the thresholds applied only to the Japanese classification. Among the 34 products with a price reduction, 4 had reductions of <2% (−0.16%, −0.46%, −0.55%, and −1.43%) and 17 had reductions of <5%; the Japanese Conditional share was therefore sensitive to the operational threshold (**Supplementary Table S4**).

### Exploratory Direct UK-Canada Population Alignment

Among the 29 products with an eligible final decision in both jurisdictions, direct population alignment was Exact for 2 (6.9%), Partial for 24 (82.8%), and Minimal or no clinically meaningful overlap for 3 (10.3%); no product was classified as Unclear. Of the 19 products assigned the same mapped category, only 1 had Exact population alignment. The 3 cases with Minimal or no clinically meaningful overlap comprised 1 adjacent-population source selection and 2 cases in which product-level aggregation across multiple indications resulted in different populations being represented by the selected UK and Canadian decisions (**Supplementary Table S6**).

### Japan-Specific Secondary Analysis of CEE-Derived Price Changes

CEE-derived price changes were available for all 43 products by the end of the price follow-up period.^23,24^ The median CEE-derived NHI drug price change was −4.28% (interquartile range [IQR], −8.18% to −0.50%), and 34/43 products (79.1%) underwent a price reduction. By additional clinical benefit status (**Supplementary Data File**), median price changes were −1.43% (IQR, −7.09% to 0.00%) for demonstrated benefit, −4.28% (IQR, −8.30% to −3.21%) for mixed findings, and −6.01% (IQR, −8.07% to −4.29%) when benefit was not demonstrated; corresponding price reductions occurred in 13/21 (61.9%), 15/16 (93.8%), and 6/6 products (100.0%), respectively (**Figure 4**). Among products that underwent a price reduction, median changes were −5.05%, −4.29%, and −6.01% in the same 3 groups, respectively.

**Figure 4.**
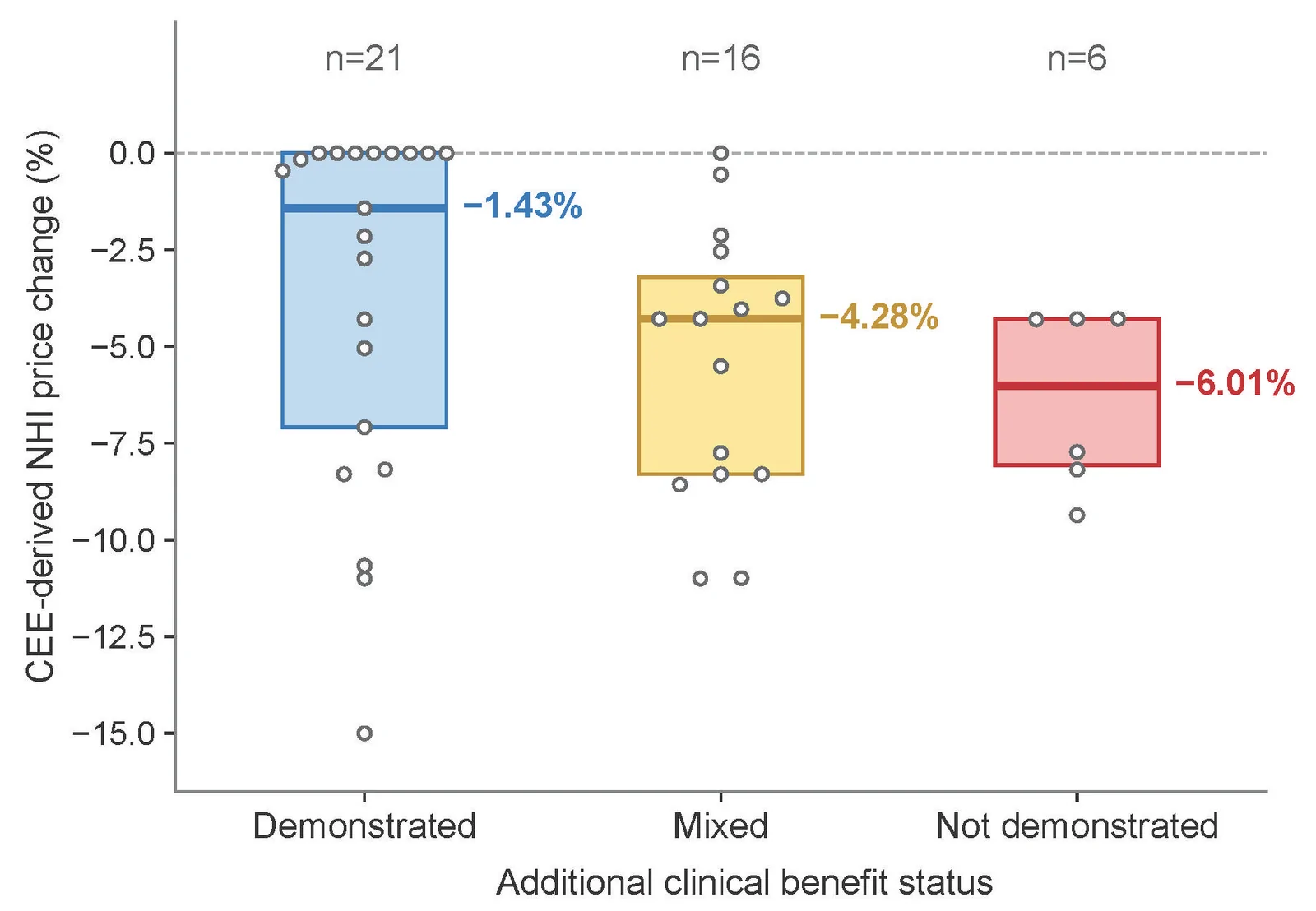
Distribution of CEE-Derived NHI Price Changes by Additional Clinical Benefit Status Each circle represents 1 product; boxes show interquartile ranges and horizontal lines indicate medians. Negative values indicate price reductions.

## DISCUSSION

To our knowledge, this is the first product-level study to use Japan’s CEE-evaluated products as the index cohort and map appraisal outputs from Japan, the UK, and Canada to common categories. We found that mapped-category agreement could coexist with differences in both the institutional meaning of the decisions and the populations appraised. In the direct UK-Canada analysis, only 1 of the 19 products with mapped-category agreement had Exact population alignment.

The higher UK-Canada mapped-category agreement should not be interpreted as a stable ranking of institutional similarity. It may reflect both the broadly coverage-oriented roles of NICE and CDA-AMC^9,18,20,25^ and the distribution of Not assessed classifications. In the complete-case analyses, which used different denominators, Japan-Canada was numerically highest (**Supplementary Table S3**). Relative to the agreement expected under independent classification, pairwise comparisons involving Japan did not consistently exceed this benchmark, whereas UK-Canada agreement exceeded it (60.5% vs 42.7%) in the main analysis and under both materiality thresholds (**Table 2, Supplementary Table S4**). Seven of the 26 concordant UK-Canada classifications were shared Not assessed classifications, indicating that part of the observed concordance reflected shared nonappraisal rather than concordant appraisal judgments. Other possible contributors, including submitted evidence, appraisal timing, reference-case methods, and other country-specific decision processes, were not examined. Japan’s CEE functions instead as a price-adjustment-oriented HTA,^2–5^ and in this cohort, Japan had no Not assessed classifications by design and no Negative classifications. The lower Japan-UK and Japan-Canada estimates may therefore partly reflect this asymmetry in the mapped category distribution rather than divergent assessments of individual products alone.

Conditional was the most frequent category in all 3 jurisdictions, but its institutional meaning differed substantially across jurisdictions (**Table 1**).^3–5,18–20^ Where recommendations within a single jurisdiction differed by target population, aggregation to a single category also risks concealing heterogeneity within the Conditional designation.

Product-level examples illustrate distinct mechanisms underlying mapped agreement and disagreement. Inclisiran was Conditional in all 3 jurisdictions, yet Canada had also issued a separate “Do not reimburse” recommendation for a different population, so complete agreement did not imply an identical appraisal question (**Supplementary Note S.N.3**). For elranatamab, the UK and Canada were Conditional whereas Japan was Positive: the Japanese ICER was below the applicable ¥7.5 million/QALY threshold, so no CEE-based price reduction was triggered, whereas the UK and Canadian recommendations carried appraisal-derived conditions. For polatuzumab vedotin, direct UK-Canada population alignment was Exact, yet the UK was Positive and Canada Conditional, because a commercial arrangement alone did not make a UK recommendation Conditional whereas the Canadian recommendation carried clinical criteria and conditions (**Supplementary Note S.N.4**).

Overall, Conditional predominance is a pattern of common-category mapping rather than evidence of qualitative similarity in the content of decisions.^26,27^ The expected-agreement and attainable-maximum benchmarks reported in **Table 2** contextualize observed agreement given the mapped category distributions. In the Japan-specific secondary analysis, median price changes and the frequency of price reduction (13/21, 15/16, and 6/6) followed the same ordering across the demonstrated, mixed, and not demonstrated categories, but the interquartile ranges overlapped substantially, the not demonstrated group comprised 6 products, and 8 of the 21 products with demonstrated benefit had no price change. Among products that underwent a reduction, the medians no longer followed that ordering, indicating that the full-cohort gradient reflects, in part, whether a price reduction occurred rather than its magnitude alone. Because the analysis was descriptive and unadjusted, it does not establish an independent or causal effect of additional clinical benefit on the magnitude of price adjustment.

Exact indication/population alignment with the Japan CEE target was rare, so agreement or disagreement may reflect differences in the clinical questions evaluated as well as in institutional decision-making. In the direct UK-Canada comparison, most populations were classified as Partial and retained clinically meaningful overlap, but Partial is a broad descriptive category and should not be read as evidence of near-equivalent decision populations. Single-product boundary-case reclassifications changed no agreement estimate by more than 2.4 percentage points (**Supplementary Table S2**), whereas analytic definitions applied across multiple products had larger effects; restricting eligible UK sources to Technology Appraisals reduced complete 3-jurisdiction agreement from 32.6% to 25.6%.

Cross-national HTA comparison studies have documented substantial variation in appraisal recommendations, including between the UK and Canada,^13,14,27–30^ attributed to differences in HTA system design, assessment practices, evidence interpretation, and deliberative processes.^9,26,27,31^ Such comparisons have generally used indication-specific appraisals or drug-indication combinations^12–14,30^; the closest study examined UK, Canadian, and Australian appraisals of orphan drugs approved in Japan without incorporating Japan’s own appraisal outputs,^10^ and prior work within Japan’s CEE program has characterized discrepancies between company-submitted and public analyses.^15^ The present study instead used a Japan-CEE-indexed product cohort, retained products without an eligible foreign appraisal, and assessed population alignment separately, so observed divergence cannot be attributed solely to differences in value judgments.

Ongoing Chuikyo review and the December 2025 ministerial budget agreement indicate continued reform of Japan’s CEE system.^8,32^ The observed pattern therefore describes price-adjustment outcomes under the current rule-based framework, which itself incorporates assessments of additional clinical benefit, rather than an independent effect of additional clinical benefit.

These findings also raise broader questions for Japan’s CEE system beyond price adjustment alone, including how strongly price-adjustment outcomes should be differentiated by additional clinical benefit while balancing incentives for timely market entry and patient access; the scope and selection criteria for designation, given that 37/43 products in this cohort were H1; whether CEE should inform listing decisions as well as post-listing price adjustment, given that products classified as Negative in the UK or Canada remained reimbursed in Japan; how severity or other modifiers should be incorporated, alongside existing higher ICER reference values for oncology, pediatric, and designated intractable or rare disease products; and the completeness and accessibility of published appraisal documentation, on which cross-national comparison depends. These are questions for future evaluation rather than implications established here.

Because CEE-derived adjustments reduce nationally published NHI prices, they may also be relevant to external pricing policies that reference Japanese prices, such as the Generating Cost Reductions for U.S. Medicaid (GENEROUS) Model, whose 8-country basket includes Japan.^7,33,34^ That model, however, sets its benchmark using manufacturer-reported net prices rather than published list prices,^35^ and whether CEE-adjusted Japanese prices influence foreign pricing was not examined here.

This study has several limitations. First, the cohort was highly selected: 37/43 (86.0%) products were designated H1, enriching it for high-sales products more likely to have been launched and appraised in other jurisdictions, so the cohort is not representative of medicines appraised in the UK or Canada and Not assessed classifications would be expected to be less frequent. The direction of the resulting bias differs by comparison: because Japan had no Not assessed classifications, fewer such classifications in the UK or Canada would remove a structural source of disagreement and could increase agreement in comparisons involving Japan, whereas in the UK-Canada comparison shared Not assessed classifications contributed to agreement; the net direction is therefore uncertain. The low frequencies of Negative classifications in the UK (2/43) and Canada (4/43) are also consistent with selection toward products more likely to receive favorable recommendations, although this cannot be established without comparison with products not selected for CEE. Second, data extraction was not independently duplicated, and appraisal selection and population alignment required judgment; residual misclassification cannot be excluded. Source selection was a consequential judgment-dependent step because the selected decision determined the mapped category. The same investigator who made the original source selections also identified the boundary cases, so selections not recognized as borderline may have been missed; accordingly, the observed changes of no more than 2.4 percentage points apply only to the boundary cases identified. However, documented mapping rules were applied, classifications and boundary cases were reviewed with the coauthors, and source details are provided in the **Supplementary Data File**. Third, the mapped categories represented operational harmonization, population alignment with the Japan CEE target was usually partial, and product-level aggregation may have obscured indication-specific differences. The UK comparator was limited to NICE and NHS England, and inclusion of NICE Guidelines and NHS England commissioning policies where no eligible TA was available affected category distribution.^18^ The direct UK-Canada analysis used decisions selected for the Japan-anchored main analysis; compared decisions were not necessarily contemporaneous, and appraisal methods and decision frameworks evolved during the study period, particularly in the UK.^36^ Fourth, simple agreement estimates were influenced by imbalanced marginal distributions and by inclusion of Not assessed as a category. Finally, public appraisal outputs may not reflect realized access or net prices; the mixed-benefit group was heterogeneous and the not-demonstrated group was small.

## CONCLUSIONS

Structured mapping of appraisal outputs for 43 Japan-CEE–evaluated products showed that mapped-category agreement across Japan, the UK, and Canada could coexist with differences in institutional meaning and the populations appraised. International comparisons using common categories should therefore account for these differences rather than interpret agreement as evidence of similarity between HTA systems. In Japan, CEE-derived price changes varied across additional clinical benefit categories. Because the current price-adjustment rules themselves incorporate assessments of additional clinical benefit, these descriptive differences characterize outcomes under those rules rather than an independent effect of additional clinical benefit.

### Disclosures

K.T. is a full-time employee of argenx Japan K.K. This study was conducted independently of that employment, and argenx had no role in the design, data collection, analysis, interpretation, or decision to submit this manuscript. A.S. and Y.N. declare no conflicts of interest.

### Ethics Approval and Consent to Participate

This study was a secondary analysis of publicly available, aggregated health technology assessment appraisal documents and published national drug pricing information. It did not involve human participants, human biological material, or individually identifiable personal data. Accordingly, institutional review board approval and informed consent were not required.

## Supporting information

Online Supplementary Material

Supplementary Data File

## Data Availability

Product-level classifications, the source-level appraisal audit trail, the direct UK-Canada alignment classifications, and the CEE-derived price outcomes followed through August 5, 2026, are provided in the **Supplementary Data File.** Additional data underlying the analyses are available from the corresponding author on reasonable request.
