# Supplementary material for: Comparison of HTA Appraisal Outcomes in Japan, the UK, and Canada: A Cross-Sectional Mapping Study
Source: J Health Econ Outcomes Res. 2026 Nov 13;13(2):231–40. doi: 10.36469/001c.172102 (PMC13770490; doi:10.36469/001c.172102)
Supplement: Supplementary file 1 [file jheor_2026_13_2_172102_377191.pdf]

Journal of Health Economics and Outcomes Research

Online Supplementary Material

Comparison of HTA Appraisal Outcomes in Japan, the UK, and Canada: A Cross-Sectional Mapping Study. *JHEOR*. 2026;13(2):231-240. doi:10.36469/jheor.2026.172102

Supplementary Tables S1-S6 1

Supplementary Notes S.N.1-S.N.5 7

Supplementary Appendix 8

This supplementary material has been provided by the authors to give readers additional information about their work.

This is an open-access article distributed under the terms of the Creative Commons Attribution 4.0 International License (CCBY-4.0). View this license's legal deed at http://creativecommons.org/licenses/by/4.0 and legal code at http://creativecommons.org/licenses/by/4.0/legalcode for more information.

Supplementary Table S1. Non-TA UK decision sources in the main analysis and their reclassification under the TA-only strict sensitivity analysis.

No.	Brand Name	UK source (main analysis)	UK category (main)	UK category (TA-only strict)
1	Trelegy 100 Ellipta	NICE NG115 (COPD guideline)	Conditional	Not assessed
9	Rybelsus	NICE NG28 (T2D guideline)	Conditional	Not assessed
12	Arikayce	NHS England Clinical Commissioning Policy 2111 (NTM)	Conditional	Not assessed
41	Briviact	NICE NG217 (Epilepsies guideline)	Conditional	Not assessed

Four products were reclassified from Conditional to Not assessed; Japan and Canada classifications were unchanged.

Abbreviations: COPD, chronic obstructive pulmonary disease; NTM, nontuberculous mycobacterial pulmonary disease; T2D, type 2 diabetes.

Supplementary Table S2. Effect of sensitivity and boundary-case scenarios on pairwise and complete three-jurisdiction mapped-category agreement (n=43).

Scenario	Classification change	JP–UK	JP–CA	UK–CA	All three
Main analysis (reference)	—	18/43 (41.9%)	22/43 (51.2%)	26/43 (60.5%)	14/43 (32.6%)
UK TA-only strict	UK: 4 products reclassified from Conditional to Not assessed (Nos. 1, 9, 12, and 41: Trelegy, Rybelsus, Arikayce, and Briviact)	14/43 (32.6%)	22/43 (51.2%)	24/43 (55.8%)	11/43 (25.6%)
Padcev exact-indication (No. 16)	UK: Positive → Not assessed (TA797 terminated)	18/43 (41.9%)	22/43 (51.2%)	26/43 (60.5%)	14/43 (32.6%)
Leqembi draft-inclusive (No. 36)	UK + CA: Not assessed → Negative (draft-equivalent Negative positions)	18/43 (41.9%)	22/43 (51.2%)	26/43 (60.5%)	14/43 (32.6%)
Mounjaro draft-inclusive (No. 28)	CA: Not assessed → Conditional (draft recommendation to reimburse with conditions)	18/43 (41.9%)	22/43 (51.2%)	27/43 (62.8%)	14/43 (32.6%)
Polivy alternative TA (TA874) (No. 11)	UK: Positive → Conditional (TA874, untreated DLBCL, used as primary instead of TA649)	17/43 (39.5%)	22/43 (51.2%)	27/43 (62.8%)	14/43 (32.6%)
Leqvio alternative population (No. 33)	CA: Conditional → Negative (nFH+ASCVD recommendation)	18/43 (41.9%)	21/43 (48.8%)	25/43 (58.1%)	13/43 (30.2%)

Abbreviations: ASCVD, atherosclerotic cardiovascular disease; CA, Canada; DLBCL, diffuse large B-cell lymphoma; JP, Japan; nFH, non-familial hypercholesterolemia; TA, Technology Appraisal.

Supplementary Table S3. Cross-jurisdictional mapped-category agreement after excluding Not assessed classifications.

Comparison	Main analysis	Excluding Not assessed
Japan–UK	18/43 (41.9%)	18/33 (54.5%)
Japan–Canada	22/43 (51.2%)	22/32 (68.8%)
UK–Canada	26/43 (60.5%)	19/29 (65.5%)
Complete three-jurisdiction agreement	14/43 (32.6%)	14/29 (48.3%)

For each pairwise comparison, products classified as Not assessed in either jurisdiction were excluded. For complete three-jurisdiction agreement, products classified as Not assessed in any jurisdiction were excluded.

Supplementary Table S4. Sensitivity of Japanese classifications and cross-jurisdictional agreement to alternative materiality thresholds for CEE-derived price reductions.

Panel A. Japanese classification

**Threshold for Conditional**	**Japan Conditional, n (%)**
Any price reduction (main analysis)	34/43 (79.1%)
Price reduction ≥2%	30/43 (69.8%)
Price reduction ≥5%	17/43 (39.5%)

Panel B. Agreement benchmarks under each threshold

**Threshold**	**Comparison**	**Observed agreement**	**Expected under independence**	**Attainable maximum**
Any reduction	Japan–UK	18/43 (41.9%)	47.5%	31/43 (72.1%)
	Japan–Canada	22/43 (51.2%)	51.5%	28/43 (65.1%)
	All three	14/43 (32.6%)	28.7%	24/43 (55.8%)
≥2% reduction	Japan–UK	16/43 (37.2%)	43.9%	31/43 (72.1%)
	Japan–Canada	20/43 (46.5%)	45.4%	28/43 (65.1%)
	All three	12/43 (27.9%)	25.4%	24/43 (55.8%)
≥5% reduction	Japan–UK	11/43 (25.6%)	31.9%	24/43 (55.8%)
	Japan–Canada	9/43 (20.9%)	25.7%	17/43 (39.5%)
	All three	4/43 (9.3%)	14.4%	17/43 (39.5%)

Abbreviation: CEE, cost-effectiveness evaluation.

Expected agreement was calculated under independent classification from the observed marginal distributions. Attainable maximum denotes the maximum agreement possible given the observed marginal distributions. The 2% and 5% thresholds were analytical materiality thresholds and are not formal thresholds in Japan's CEE framework. UK–Canada classifications were unaffected by the Japanese materiality threshold; observed agreement remained 26/43 (60.5%), expected agreement under independence 42.7%, and the attainable maximum 36/43 (83.7%).

Supplementary Table S5. Characteristics of the 43 included products.

**Characteristic**	**n**	**%**
CEE designation category		
H1 (predicted peak annual sales ≥ JPY 10 billion)	37	86.0
H2 (predicted peak annual sales ≥ JPY 5 billion and < JPY 10 billion)	5	11.6
H3 (exceptionally high reimbursement price, added indication, or material new evidence)	1	2.3
Orphan / intractable disease designation in Japan		
Designated (orphan and/or intractable)	12	27.9
Not designated	31	72.1
Therapeutic area		
Oncology	12	27.9
Neurology	6	14.0
Metabolic / endocrine	5	11.6
Infectious diseases	5	11.6
Hematology (non-malignant)	4	9.3
Respiratory	3	7.0
Dermatology	3	7.0
Nephrology	2	4.7
Other (psychiatry, cardiology, gastroenterology)	3	7.0

Abbreviations: CEE, cost-effectiveness evaluation; JPY, Japanese yen.

Supplementary Table S6. Final-decision comparability and direct UK–Canada population alignment.

Panel A. Final-decision comparability among all 43 products

Final-decision comparability	n	%
Comparable—eligible final decision in both jurisdictions	29	67.4
Not comparable—neither jurisdiction had an eligible final decision	7	16.3
Not comparable—UK only lacked an eligible final decision	3	7.0
Not comparable—Canada only lacked an eligible final decision	4	9.3
Total	43	100.0

Panel B. Direct UK–Canada population alignment among comparable products, by mapped-category agreement (n=29)

Direct population alignment	Same mapped category, n	Different mapped category, n	Total, n (%)
Exact	1	1	2 (6.9)
Partial	16	8	24 (82.8)
Minimal or no clinically meaningful overlap	2	1	3 (10.3)
Unclear	0	0	0 (0.0)
Total	19	10	29 (100.0)

Comparability was assessed for all 43 products; direct population alignment was evaluated only for the 29 products with an eligible final decision in both jurisdictions. These 29 products correspond to the UK–Canada complete-case set in Supplementary Table S3. Product-level classifications and rationales are provided in Supplementary Data File. The three Minimal or no clinically meaningful overlap cases were enfortumab vedotin (Padcev), daratumumab/hyaluronidase (Darzquro), and selpercatinib (Retevmo).

Supplementary Notes

S.N.1 No. 16 Enfortumab vedotin (Padcev) — Terminated exact-match appraisal and closest eligible final decision

NICE TA797 matched the Japan CEE target but was terminated after no company submission and was therefore ineligible. TA1097, the eligible final NICE decision with the greatest clinically meaningful overlap, was selected as Closest available and classified as Positive. Under exact-indication matching, the UK was Not assessed; agreement estimates were unchanged.

S.N.2 No. 36 Lecanemab (Leqembi) — Final-only: Not assessed (main analysis) / Negative (draft-inclusive boundary-case assessment)

No final NICE or CDA-AMC decision had been published by the May 31, 2026 data cutoff. The latest UK draft position was not recommended, and the Canadian draft recommendation was "Do not reimburse." Both jurisdictions were therefore classified as Not assessed in the main analysis and as Negative in the draft-inclusive boundary-case assessment.

S.N.3 No. 33 Inclisiran (Leqvio) — Mixed decisions under single category

CDA-AMC recommended reimbursement with clinical criteria and/or conditions for heterozygous familial hypercholesterolemia (HeFH) but not reimbursement for non-familial hypercholesterolemia with atherosclerotic cardiovascular disease (nFH + ASCVD). Neither recommendation covered the full product-level Japan CEE target; because overlap was judged similar, the more recent HeFH recommendation was selected as the tie-breaker, yielding a Canadian classification of Conditional. The nFH + ASCVD recommendation was retained as the boundary-case alternative.

S.N.4 No. 11 Polatuzumab vedotin (Polivy) — Primary appraisal selection between two NICE Technology Appraisals

The Japan CEE evaluated relapsed or refractory diffuse large B-cell lymphoma (DLBCL), corresponding to NICE TA649 rather than TA874, which addressed untreated DLBCL. TA649 was therefore selected and classified as Positive because its commercial arrangement alone did not constitute an appraisal-derived clinical restriction. In the exploratory direct UK–Canada analysis, the selected decision populations were classified as Exact, but the mapped categories differed: UK Positive versus Canada Conditional. TA874 was retained as the boundary-case alternative.

S.N.5 No. 28 Tirzepatide (Mounjaro) — Final-only: Not assessed (main analysis) / Conditional (draft-inclusive boundary-case assessment)

CDA-AMC had issued only a draft recommendation to reimburse tirzepatide with clinical criteria and/or conditions by the May 31, 2026 data cutoff. Canada was therefore classified as Not assessed in the final-only main analysis and as Conditional in the draft-inclusive boundary-case assessment (Supplementary Table S2).

Supplementary Appendix. Structured data-extraction form.

The fields below reproduce the structured information recorded for appraisal sources, population alignment, and the Japan-specific secondary analysis. Analytical calculations and aggregate summary measures derived from these recorded fields are not reproduced as data-extraction fields. Permitted values/format indicate the coding conventions used in the study; not all permitted values were observed in the recorded data. In the "Corresponding field in Supplementary Data File" column, "Audit Trail" refers to the "Appraisal Source Audit Trail" sheet and "Direct Alignment" to the "UK–Canada Direct Alignment" sheet.

Section 1. Record identifiers

**Field**	**Definition / recording instruction**	**Permitted values / format**	**Corresponding field in Supplementary Data File**
No.	Sequential product identifier shared across all sheets; links each source record to a product.	Integer, 1-43	Audit Trail, col. A
Brand name	Brand name of the product under which the Japan CEE was conducted.	Free text	Audit Trail, col. B
Generic name	Generic name(s) of the active substance(s), as recorded in the study dataset.	Free text	Audit Trail, col. C
Jurisdiction	Jurisdiction to which the source record applies.	"Japan"; "UK (England)"; "Canada"	Audit Trail, col. D
Japan CEE target indication	Indication evaluated under Japan's CEE, recorded as the reference against which UK and Canadian appraisal populations were assessed.	Free text	Audit Trail, col. E

Section 2. Appraisal source and mapping

Field	Definition / recording instruction	Permitted values / format	Corresponding field in Supplementary Data File
Source organization	Agency issuing the document. For Canada, record CADTH for decisions before May 2024 and CDA-AMC thereafter; record “CADTH / CDA-AMC” where no decision date was available.	Recorded values: “NICE”; “NHS England”; “NICE / NHS England”; “CADTH”; “CDA-AMC”; “CADTH / CDA-AMC”; “C2H (NIPH)”; “C2H (NIPH) / MHLW (Chuikyo)”	Audit Trail, col. F
Document type	Document class and, where applicable, the issuing committee.	Free text; recorded examples: “Technology appraisal (NICE TA)”; “Clinical guideline (NICE)”; “NHS England Clinical Commissioning Policy”; “pERC Final Recommendation”; “CDEC Final Recommendation”; “CEE public analysis result / price adjustment decision”	Audit Trail, col. G
Document ID	Agency-assigned project or document identifier. Leave blank where none was assigned or none could be confirmed from the publicly available document; do not enter a placeholder.	Free text, or blank	Audit Trail, col. H
Document title	Title of the document as published.	Free text	Audit Trail, col. I
Publication/ decision date	Date of the final recommendation, final guidance, or other formal decision used for the record; for a supporting document without a separate decision date, record its publication date. For Canadian final-recommendation records, record the final recommendation date printed on the recommendation document, not the later posting date of the corresponding journal record.	Date	Audit Trail, col. J

Field	Definition / recording instruction	Permitted values / format	Corresponding field in Supplementary Data File
Status at cutoff	Status of the source document at the cohort/appraisal cutoff (May 31, 2026).	“Final”; “Draft”; “Ongoing”; “Deferred”; “Terminated”; “Superseded”; “Not evaluated”	Audit Trail, col. K
Decision wording / classification basis	Operative wording of the decision, recorded as the basis on which the mapped category was assigned.	Free text	Audit Trail, col. L
Indication/ population alignment	Alignment of the appraisal population with the Japan CEE target indication and population. Judged across seven dimensions (disease or histologic subtype, biomarker, severity or disease activity, age, prior treatment, line of therapy, and treatment regimen), which were also used for the direct UK–Canada comparison. Recorded as an overall category rather than dimension by dimension. Japan records are Exact by construction, the Japan CEE target being the reference.	“Exact”; “Partial”; “Closest available”; “No match / Not evaluated”	Audit Trail, col. M
Source role	Role of the record in the analysis. Only “Primary – main classification” records determined the mapped categories reported in the manuscript.	“Primary – main classification”; “Supporting – indication-specific evidence”; “Supporting – CEE analysis / indication-specific evidence”; “Supporting – superseded historical decision”; “Boundary/sensitivity”	Audit Trail, col. N

Field	Definition / recording instruction	Permitted values / format	Corresponding field in Supplementary Data File
Mapped category	Common appraisal category to which the source record was mapped under the study rules. For records with the source role "Primary – main classification," this category determined the product-jurisdiction classification used in the main analysis; supporting, superseded, and boundary/sensitivity records did not determine the main-analysis classification.	"Positive"; "Conditional"; "Negative"; "Not assessed"	Audit Trail, col. O
Mapping rationale	Reason the recorded decision wording was mapped to the assigned category.	Free text	Audit Trail, col. P
Sensitivity/ boundary note	Any source-selection, decision-status, or other boundary-case issue relevant to the sensitivity assessment, including alternative interpretations that could change the mapped category.	Free text; blank where none applies	Audit Trail, col. Q
URL	Location of the source document. Blank where no eligible appraisal or completed reimbursement-review document was available for that product-jurisdiction pair at the cutoff.	URL, or blank	Audit Trail, col. R
Accessed date	Date on which the source was most recently accessed; where several sources are cited in one record, the latest access date.	Date	Audit Trail, col. S

Section 3. Direct UK–Canada population alignment

Field	Definition / recording instruction	Permitted values / format	Corresponding field in Supplementary Data File
Final-decision comparability	Whether an eligible final decision was available in both jurisdictions. Draft documents, terminated appraisals, and review scopes were not substituted for eligible final decisions.	"Comparable"; "Not comparable"	Direct Alignment, col. G
UK decision population	Population addressed by the selected UK decision, as stated in that decision.	Free text; blank for products classified as Not comparable	Direct Alignment, col. H
Canada decision population	Population addressed by the selected Canadian decision, as stated in that decision.	Free text; blank for products classified as Not comparable	Direct Alignment, col. I
Disease / histologic subtype	Dimension-level comparison of the two decision populations.	"Same"; "Different"; "Not applicable" (recorded as "N/A"); "Unclear"	Direct Alignment, col. J
Biomarker	Dimension-level comparison of the two decision populations.	"Same"; "Different"; "Not applicable" (recorded as "N/A"); "Unclear"	Direct Alignment, col. K
Severity / disease activity	Dimension-level comparison of the two decision populations.	"Same"; "Different"; "Not applicable" (recorded as "N/A"); "Unclear"	Direct Alignment, col. L
Age	Dimension-level comparison of the two decision populations.	"Same"; "Different"; "Not applicable" (recorded as "N/A"); "Unclear"	Direct Alignment, col. M
Prior treatment	Dimension-level comparison of the two decision populations.	"Same"; "Different"; "Not applicable" (recorded as "N/A"); "Unclear"	Direct Alignment, col. N
Line of therapy	Dimension-level comparison of the two decision populations.	"Same"; "Different"; "Not applicable" (recorded as "N/A"); "Unclear"	Direct Alignment, col. O
Treatment regimen	Dimension-level comparison of the two decision populations.	"Same"; "Different"; "Not applicable" (recorded as "N/A"); "Unclear"	Direct Alignment, col. P

Field	Definition / recording instruction	Permitted values / format	Corresponding field in Supplementary Data File
Direct population alignment	Overall alignment of the two decision populations, judged from the dimension-level comparisons.	"Exact"; "Partial"; "Minimal or no clinically meaningful overlap"; "Unclear". Recorded as "Not assigned" where one or both jurisdictions lacked an eligible final decision	Direct Alignment, col. Q
Source-selection flag	Source-selection feature distinct from population alignment, recorded using a fixed vocabulary.	"Class-level UK source"; "Population-specific Canadian source"; "Product-level multi-indication aggregation"; "Multi-indication target with indication-specific primaries"; "Closest-available UK source"; blank where none applies	Direct Alignment, col. R
Rationale	Clinically material difference or differences determining the overall alignment judgment, or a statement that none was identified.	Free text; blank where no alignment category was assigned	Direct Alignment, col. S

Dimension-level judgments were recorded as Same where the same criterion applied, Different where a clinically meaningful difference was present, Not applicable where the dimension could not logically apply, and Unclear where the dimension could apply but the available documents did not permit a judgment. Recorded values are provided in Supplementary Data File.

Section 4. Japan-specific secondary-analysis fields

**Field**	**Definition / recording instruction**	**Permitted values / format**	**Corresponding field in Supplementary Data File**
Additional clinical benefit (product level)	Product-level status derived from analysis-population-level additional clinical benefit findings extracted from C2H public analysis documents: Demonstrated if demonstrated in all evaluated populations; Not demonstrated if not demonstrated in any; Mixed if demonstrated in some but not all evaluated populations, including cases in which remaining populations were not assessable; and Not assessable if the C2H result was unavailable or inconclusive.	"Demonstrated"; "Mixed"; "Not demonstrated"; "Not assessable"	Audit Trail, col. T; Product Summary, col. N
Price basis (strength/package presentation)	Strength and package presentation to which the recorded NHI prices apply; the same presentation was used before and after the CEE-based adjustment.	Free text	Product Summary, col. O
Pre-adjustment NHI price (JPY)	NHI price immediately before the CEE-based adjustment, as reported in Chuikyo and MHLW materials.	Numeric (JPY)	Product Summary, col. P
Post-adjustment NHI price (JPY)	NHI price immediately after the CEE-based adjustment, as reported in Chuikyo and MHLW materials.	Numeric (JPY)	Product Summary, col. Q
